# Every Person Counts in a Fair Transition to Net Zero: A UK Food Lens Towards Safeguarding Against Nutritional Vulnerability

**DOI:** 10.1111/nbu.70032

**Published:** 2025-10-16

**Authors:** A. Spiro, L. Bardon, J. Fanzo, Z. Hill, S. Stanner, M. H. Traka

**Affiliations:** ^1^ British Nutrition Foundation London UK; ^2^ Quadram Institute Norwich UK; ^3^ Columbia University New York USA

## Abstract

The British Nutrition Foundation and Quadram Institute hosted a multidisciplinary roundtable, chaired by Professor Jessica Fanzo, to explore how the UK food system can be transformed to achieve net zero targets while ensuring nutritional adequacy, food security, and health equity across the life course. Current dietary patterns are significant contributors to the global burden of chronic disease, while food systems also cause considerable environmental harm. Agriculture, as both a major driver of climate change and a sector highly vulnerable to its effects, plays a crucial role in shaping both environmental change and food security. In the UK, dietary patterns often diverge from established guidelines, particularly among vulnerable groups, highlighting a food environment that fails to promote nutritional security or support balanced, sustainable, and diverse plant‐rich diets for long‐term health. Achieving a shift towards healthier, more sustainable diets requires a collaborative, cohesive, interdisciplinary, and innovative approach that integrates both nutritional and environmental goals across the entire food system. Roundtable participants considered how targeted action from policymakers, industry, and the agricultural sector can support this transition without compromising nutritional security. Participants emphasised that strategies to promote plant‐rich diets must account for population‐specific nutritional requirements and socioeconomic constraints. A key concern was ensuring that the transition to net zero does not exacerbate existing dietary inequalities. The discussion highlighted vulnerable groups, such as children, adolescents, pregnant women, and older adults, who may be at greater risk of nutritional inadequacies, particularly for vitamin B12, iron, and iodine, as efforts to reduce reliance on animal‐based foods accelerate. Ensuring access to affordable, nutrient‐dense, and bioavailable alternatives is crucial. The significant role of the private sector (manufacturers, retailers and out‐of‐home providers) in shaping the food environment was acknowledged, with an emphasis on the need for greater accountability. Participants called for robust regulatory policies to level the playing field and incentivise the production and promotion of healthier, more sustainable foods. Whilst the use of the terms ‘high in fat, sugar or salt’ (HFSS) and ‘ultra‐processed foods’ (UPF) formed part of the discussion, particularly concerning processed plant‐based alternatives, the primary message was to use such frameworks as tools to drive broader food system transformation, rather than distractions from the ultimate goal of enabling dietary patterns that are both health‐promoting and environmentally sustainable.

## 1 Why Do We Need Urgent Change?

Foods, diets and nutritional status are critical contributors to the global burden of disease, while current food systems cause substantial environmental damage. To avert potentially catastrophic outcomes, transformation is urgently needed to address climate change and enhance global health and nutrition security (Willett et al. 2019). This transformation will involve dietary changes, reductions in food losses and waste, and improvements in global agricultural practices and efficiency.

The triple burden of malnutrition: overweight and obesity, undernutrition, and micronutrient deficiencies is a global concern (Nature Food 2023). Diets have been identified as a significant risk factor for mortality and morbidity, with 11 million deaths (22% of deaths among adults) and 255 million disability‐adjusted life years (15% of those among adults) attributable to dietary risk factors, in particular high intake of sodium (salt) and low intake of whole grains and fruit (Afshin et al. 2019; Willett et al. 2019). This makes poor diet the third highest risk factor for the global burden of disease, after high blood pressure and tobacco use (Afshin et al. 2019). In 2022, the World Health Organization (WHO 2024) reported that 2.5 billion adults were overweight, including 890 million who were living with obesity, while 390 million were underweight. Furthermore, 69% of women of reproductive age (15–49 years) were affected by one or more micronutrient deficiencies (Stevens et al. 2022). The developmental, economic, social, and medical impacts of poor nutrition have a serious and lasting effect on individuals, families, communities, and countries.

Simultaneously, food systems, including production and value chains, processing and distribution, have a profound influence on climate change, although the extent of the impact depends on where foods are grown and the type of system. Global food systems are responsible for around a third of global greenhouse gas (GHG) emissions, including carbon dioxide (CO_2_), methane (CH_4_), and nitrogen dioxide (NO_2_) (Crippa et al. 2021). Agriculture is a significant driver of habitat and biodiversity loss and soil degradation, and accounts for 70% of total freshwater withdrawals (Ritchie and Roser 2024). Concerns also exist regarding animal welfare within farming, including intensive management that can foster disease emergence (Verkuijl et al. 2024; Hayek 2022). Practices associated with intensive farming systems can be harmful to biodiversity, including monoculture, under‐rotation, overuse of fertilisers and pesticides, and heavy mechanisation (EC 2023), and health (Smit and Heederik 2017).

There is an urgent need to act both on climate change mitigation and adaptation to climate change. Climate change mitigation refers to actions required to reduce GHG emissions to address the underlying causes of climate change, whereas adaptation refers to actions required to manage the effects of climate change (Burnett 2024). Yet consideration of how climate change can adversely impact the accessibility and affordability of a healthy diet may be less recognised. Food production systems, including the quality, quantity, and supply of food, and the livelihoods of food producers, are highly vulnerable to climate change (Tchonkouang et al. 2024; Beach et al. 2019). Climate change manifests in fires, excessive temperatures, flooding, increased pest prevalence, decreased pollinators, poor livestock adaptation, higher food prices, and increased poverty, all of which have serious implications for food and nutrition security (FAO, IFAD, UNICEF, WFP & WHO. 2024; Godde et al. 2021; Nelson et al. 2016). Therefore, there is a pressing need for a shift towards more sustainable food systems that not only address global food production and consumption but ensure resilience in the face of climate change.

Climate change, obesity, and undernutrition are often viewed as separate concerns, but they are closely interrelated. From a nutrition and public health perspective, there is increasing consensus on the types of foods that can negatively impact the climate, such as high intake of red and processed meat (Poore and Nemecek 2018; BDA 2020; Steenson and Buttriss 2021). Plant‐rich diets that include higher quantities of vegetables, pulses (beans/lentils), fruit, whole grains, nuts, and seeds, as exemplified by healthy eating guidelines, such as the Eatwell Guide and the Mediterranean dietary pattern, are widely recognised for their health and environmental benefits (Shannon et al. 2024). These patterns advocate for moderating animal‐based foods and eating fewer products high in saturated fat, salt, or sugar. However, current diets do not meet these guidelines; for example, in the UK, less than 1% of the population adheres to all nine of the dietary guidelines (Scheelbeek et al. 2020). Whilst food‐based dietary guidelines promote healthier and more sustainable dietary patterns, more substantial changes may be necessary to drive the transition towards a sustainable global food system by 2050. The influential EAT‐Lancet *Planetary Health Diet*, a reference dietary pattern designed to promote human health whilst remaining within planetary boundaries (Willett et al. 2019), recommended a more stringent shift in dietary patterns (Willett et al. 2019) with a series of targets and limits for specific food groups, with smaller contributions from animal‐sourced proteins and dairy foods. Whilst receiving recognition for its potential to inform policy (Singh Chawla 2024), improve public health (Karavasiloglou et al. 2023), and stimulate public awareness around transitioning to sustainable diets (Tulloch et al. 2023), the EAT‐Lancet *Planetary Health Diet* also faced criticism for failing to adequately consider the economic, social, and cultural diversity aspects of diets (Tulloch et al. 2023; Hirvonen et al. 2020). Some concerns have also been raised over the adequacy of the planetary health diet for certain essential micronutrients that are commonly insufficient worldwide, such as calcium, iron, and zinc (Beal et al. 2023; Young 2022); particularly for population subgroups with increased nutrient needs (Beal et al. 2023). Consequently, the EAT‐Lancet Commission 2.0 (due to report in October 2025) was convened to review the latest evidence on defining and quantifying a healthy reference diet, with an added focus on diversity, equity, and justice (EAT‐Lancet Commissioners 2023; Bereza et al. 2023). The Commission is also undertaking consultations to better understand implementation challenges and address concerns such as micronutrient adequacy.

Our current food system has been widely described as ‘broken’ (House of Lords Food, Diet and Obesity Committee 2024), with a clear and urgent need for change. UK data suggests that on average population dietary patterns are not meeting recommendations, with low intake of fruit and vegetables and fibre and high intake of saturated fat and free sugars. For example, for children aged 11 to 18 years and for adults, 96% did not meet the fibre recommendation, and only 9% of children aged 11 to 18 years and 17% of all adults met the 5 A Day recommendation (OHID 2025). In addition, the accessibility and affordability of healthier diets are not experienced equally; the most deprived communities are affected disproportionately by higher rates of diet‐related ill health, such as obesity, type 2 diabetes, cardiovascular disease and dental decay (UK Parliament 2022).

The transition to net zero offers an opportunity to reshape the current food environment, and there is growing recognition across government, local authorities, industry, and wider society of the urgent need to support healthier and more sustainable diets. However, a just transition must be equitable and not unduly burden those who are already vulnerable. These groups face restricted choices and have less ability to control their environment, adapt their behaviours, or respond to new risks, making them particularly vulnerable to the effects of climate and economic shocks and diet‐related health concerns.

The Quadram Institute and the British Nutrition Foundation (BNF) convened a roundtable in May 2024 to provide a forum for open discussion on the journey towards healthier and more sustainable diets, considering some of the social, nutritional, and environmental issues during the transition to net zero (i.e., the commitment to reducing greenhouse gas emissions, as drivers of climate change, to net zero by 2050). The event considered:

Current intakes in relation to government dietary recommendations and any potential unintended consequences for nutritionally vulnerable groups during the transition to net zero.

How responses from policymakers, industry stakeholders, and consumers can safeguard nutritional security.

A key discussion point was the use of a cross‐disciplinary social impact lens to ensure that the transition to a healthy, climate‐smart food system is not only just and sustainable but also socially acceptable. In essence, as we strive to solve environmental sustainability challenges in our food systems, it is crucial that we do not create or exacerbate issues such as diet and health disparities and social inequalities or shift environmental harms to other regions or populations.

A select group of thought leaders and experts from across the food chain, with expertise in sustainability, nutrition, vulnerable groups, and agriculture, were invited to participate. This group considered nutrients, with a focus on micronutrients, and populations of concern and discussed how stakeholders can address the challenges from consumer, policy, and farm‐to‐fork perspectives on some of the social, nutritional, and environmental issues. The roundtable was chaired by Professor Jessica Fanzo, Professor of Climate and Director of the Food for Humanity Initiative at the Columbia Climate School at Columbia University.

## 2 Vulnerable Groups and Nutrients of Concern

Specific population subgroups are more vulnerable to inadequate intake and diet‐related health concerns. Modelling studies suggest that adjusting current diets to become both healthy and sustainable requires significant shifts in the types of foods and quantities currently consumed by the majority of the population (Horgan et al. 2016). Importantly, the feasibility of such adjustments for nutritionally vulnerable populations has not been adequately considered. Policies aimed at promoting healthier and more sustainable diets should be designed to avoid exacerbating dietary inequalities, particularly for households experiencing food insecurity, lower socio‐economic groups, and life stages at higher risk of poor dietary outcomes. Nutritional needs evolve throughout the life course; certain life stages may increase the risk of nutrition vulnerability, for example, periods of growth when nutrient requirements are increased, or older age when food intake may decrease.

### 2.1 Dietary Inequalities

Unacceptable disparities exist between socio‐economic and health status (OHID 2022). People living in deprived areas are disproportionately affected by childhood and adult obesity, childhood dental decay, and non‐communicable diseases, the risks of which can be reduced through diet and other lifestyle behaviours (POST 2022; NHS Digital 2023). Gaps in accessing healthcare, a greater burden of disease, and lower life expectancies have all been well documented for lower socio‐economic groups. Health behaviours, nutrition, income, education, housing, and access to services all influence the development of health disparities (Office for Health Improvement and Disparities 2022). Therefore, during our dietary transition towards a more sustainable diet, it is essential that extra consideration is given to reducing rather than aggravating existing health inequalities (Goldblatt et al. 2024).

The cost‐of‐living crisis (and the ongoing financial struggle), exacerbated by the COVID‐19 pandemic, the war in Ukraine, and Brexit (the United Kingdom's exit from the European Union), has increased food insecurity, disproportionately impacting the most vulnerable households, including frail older adults, children, and people with pre‐existing health conditions and disabilities (Meadows et al. 2024; House of Commons 2023; Green et al. 2021; Katikireddi et al. 2021). For many, this has meant nutrient‐dense foods are less affordable and a restriction of food choice. Low‐income or lower socioeconomic households tend to have a lower diet quality (Miller et al. 2016; Buttriss 2019), may have difficulty affording and accessing the government‐recommended healthy diet (*Eatwell Guide*), and are most at risk of micronutrient inadequacies (Miller et al. 2016; Buttriss 2019; Magee and McCann 2019). For example, lower‐income groups in the UK have lower fruit and vegetable consumption (Goudie 2023). The Food Foundation estimates that households with children in the poorest fifth of the UK population would need to spend 70% of their disposable income to meet the Eatwell Guide's recommendations (The Food Foundation 2025).

In 2022/23, 17% of children, 11% of working‐age adults, and 3% of pensioners were in food insecure households (Francis‐Devine 2024). Household food insecurity has been mirrored by a rise in food bank use. In 2022/2023, 3% of the UK population had used a food bank in the previous 12 months. The Trussell Trust, a food bank charity estimated to run around 60% of food banks in the UK, reported that between April and September 2023, it provided 1.5 million emergency food parcels, and 65% of these parcels were for families with children (Francis‐Devine 2024).

### 2.2 Nutrients of Concern

In the transition to net zero, the group recognised the health and environmental benefits of balanced and diverse plant‐rich diets, including increased intakes of fibre and some micronutrients such as vitamin C and folate (Key et al. 2022). However, the feasibility of adopting such diets at the population level remains uncertain, particularly in terms of acceptability, availability, accessibility, and affordability of nutrient‐dense plant foods (Tobi et al. 2023). Additionally, concerns have been raised, particularly among vulnerable groups with poor dietary patterns, with regards to intake of micronutrients (Leonard et al. 2024; Lin et al. 2021) that are typically present in higher quantities and/or more bioavailable forms in animal‐sourced foods, and absent or less bioavailable in unfortified plant sources. During the roundtable, three key micronutrients, vitamin B12, iodine, and iron, were specifically discussed. Protein quality was discussed as a potential concern in vulnerable older people.

#### 2.2.1 Vitamin B12

Vitamin B12 has many roles in the body, contributing to the reduction of tiredness and fatigue, and to normal function of the immune and nervous systems, psychological function, red blood cell formation, and energy‐yielding metabolism (EFSA Panel on Dietetic Products Nutrition and Allergies 2009, 2010c). Symptoms of vitamin B12 deficiency include fatigue, anaemia, peripheral neuropathy, gait disorders, ataxia (disorders that affect co‐ordination, balance, and speech), paraesthesia (pins and needles), depression, altered cognition (including delirium), and dementia (Wolffenbuttel et al. 2019). Sparse data exist on current prevalence rates of dietary vitamin B12 deficiency in the UK, but rates appear to be low. Pernicious anaemia (an autoimmune disorder) is the most common cause of vitamin B12 deficiency in the UK and is unrelated to diet (NICE 2024a, 2024b). Blood analysis data suggest that 2% of children aged 4–10 years) and 3% of adults are deficient in vitamin B12 (%, below 150 pmol/L^2^) (OHID 2025). However, identifying B12 deficiency through laboratory methods can be prone to inaccuracies (Harrington et al. 2025); thus, definitive data on vitamin B12 deficiency may be lacking.

Animal sources, such as meat, seafood, offal, eggs and dairy products, are good sources of vitamin B12 (Public Health England 2021). However, there are no reliable naturally occurring plant sources of the vitamin, and research has shown that individuals adhering to a vegan diet exhibit lower vitamin B12 status compared to non‐vegans (Gilsing et al. 2010). Individuals who avoid all foods derived from animals (e.g., following a vegan diet) are at risk of deficiency, unless they are consuming B12‐fortified foods, such as fortified breakfast cereals, fortified plant alternatives to milk and yeast extract, or taking B12 supplements. In 2022, nearly half of the plant‐based dairy milk alternatives available on the UK market were fortified with vitamin B12 (Zhang et al. 2024). Cases of clinical deficiency are rare, but they have been reported in those consuming restrictive vegan diets, particularly in babies and young children, highlighting the importance of ensuring adequate vitamin B12 intake for pregnant and lactating vegan women (Agrawal and Nathani 2009; Dubaj et al. 2020; Kiely 2021).

#### 2.2.2 Iron

Iron is an essential micronutrient that has a role in the process of cell division, contributes to the normal formation of red blood cells and haemoglobin, cognitive function, reduction of tiredness and fatigue, and normal function of the immune system (EFSA Panel on Dietetic Products Nutrition and Allergies 2010b, 2016, 2013).

Dietary sources of iron include red meat, pulses, nuts, fortified breakfast cereals, and bread. Non‐wholemeal flour must be fortified with iron (as well as calcium, thiamine, and niacin) in the UK. Despite a lower iron contribution from meat compared to cereals (see Table 1), it is well established that iron from meat (haem iron) is significantly more bioavailable than iron from plant sources (non‐haem iron), with absorption rates estimated to be two to six times higher (SACN 2010). Iron absorption and bioavailability are influenced by iron status and the presence of iron enhancers and inhibitors in the diet. Calcium, as well as non‐nutrient compounds within plants, such as certain polyphenols, phytates, and to a lesser extent oxalic acid, interact with iron and can inhibit iron absorption within the body (Piskin et al. 2022). Reducing red and processed meat consumption to within the UK dietary recommendations (i.e., individual intake limited to not more than 70 g per day) could result in significant progress towards climate change targets (FSS 2024) and would have little impact on the proportion of the adult population with low iron intakes (SACN 2010). Whilst the UK *National Diet and Nutrition Survey* (NDNS) 2019 to 2023 intake data reports average consumption of red and processed meat below 70 g per day in all age and sex groups, a wide range of intakes for red and processed meat has been reported; for example, on average, men aged 19 to 64 years ate 66 g per day, but over a quarter (27%) were high consumers (ate more than 90 g per day) (OHID 2025). We also need to consider vulnerable groups at greater risk of iron deficiency (e.g., women of child‐bearing age); if consumption in these groups is reduced, it is important to support nutritional adequacy so that any resulting decrease in micronutrient intakes is mitigated by ensuring appropriate dietary substitutions, whilst also considering bioavailability.

**TABLE 1:** Contribution of cereal and meat food group to UK dietary iron intake from UK National Diet and Nutrition Survey.

Food group	UK National Diet and Nutrition Survey 2016/2017–2018/2019 (PHE 2020)					
	1.5–3 years	4–10 years	11–18 years	19–64 years	65–74 years	75+ years
	%	%	%	%	%	%
Cereals and cereal products	52	53	50	38	37	45
Meat and meat products	12	14	19	19	17	17

Iron deficiency anaemia is common worldwide (Gardner et al. 2023) and is recognised as a public health concern by the World Health Organization (WHO 2023a, 2023b). Iron requirements increase during periods of growthand development (Safiri et al. 2021), so children, adolescents, and pregnant women are at increased risk of deficiency, as are women of reproductive age with menstrual periods, especially if heavy or prolonged (Abbaspour et al. 2014). The *NDNS* reports low iron intakes in almost half (49%) of adolescent girls (11–18 years) and 34% of adult women (19–64 years) (OHID 2025), with adolescent females in the lowest income groups at greater risk of inadequate iron intake (Thomas et al. 2023) and pregnant women of Asian, African, or Caribbean ethnicities over twice as likely to have anaemia compared to their European counterparts (Churchill et al. 2022).

The Scientific Advisory Committee on Nutrition (SACN)^1^ time trend analysis of *NDNS* data (years 2008–2019) for children aged 18 to 36 months indicated a downward trend in iron intake, equivalent to a reduction of 0.8 mg over the 11‐year period. This observation raises concerns for this age group, particularly in light of the growing shift towards more plant‐based diets (SACN 2023a). The SACN report also highlighted that children from lower socio‐economic groups, as well as certain ethnic minority groups (Asian or Asian British and Black or Black British) are at increased risk of insufficient iron intake (SACN 2023a).

#### 2.2.3 Iodine

Iodine is used in vitro to produce thyroxine, which modulates metabolism and has a role in bone and nerve development during pregnancy and infancy. Consequences of iodine deficiency include swelling of the thyroid gland, increased risk of birth defects, impaired cognitive function, and thyroid dysfunction (EFSA Panel on Dietetic Products Nutrition and Allergies 2010a). Sufficient iodine is particularly important during pregnancy to reduce the risk of complications, both during pregnancy and at birth, as well as to prevent negative health outcomes for the foetus, including birth defects and perinatal mortality (Zimmermann 2009). The richest dietary sources of iodine include seafood and fish, dairy products, and eggs (Public Health England 2021). Seaweed is a naturally rich source of iodine, but its content is highly variable, and it can provide excessive quantities, making it an unreliable and not generally recommended source (Eveleigh et al. 2020).

The 2014 SACN position statement on *Iodine and Health* identified teenage girls, low dairy consumers, those following a vegan diet, and those living with milk allergy, lactose intolerance, or fish allergy, as well as ethnic minority groups that do not consume milk and milk products, as at risk of deficiency (SACN 2014). Whilst many countries have mandatory salt iodisation policies, access to, and uptake of, iodised salt in the UK is low and is not formally recommended due to general concerns about salt intake (Bath et al. 2014; Zimmermann and Andersson 2021). A related approach is the mandatory use of iodised salt in processed foods that are consumed by a large proportion of the population. This approach has been taken in Australia with the mandatory use of iodised salt in the manufacture of bread (TLD Endocrinology 2016).

Surveys indicate that although the frequency of use is increasing, iodine is rarely used as a fortifying nutrient in plant‐based alternatives to iodine‐containing animal‐derived foods. For example, out of 1485 plant‐based products fortified with at least one nutrient on the UK market in 2022 (products from 11 food groups including milk and cream, ready meals, yogurts, other drinks, ‘meat’, for example, alternatives to deli meats, bacon and sausages, snacks, ice cream, and sauces), only 12 were fortified with iodine (Zhang et al. 2024), whilst for milk alternatives specifically, another study reported only 28% (*n* = 29) were fortified with iodine (Nicol et al. 2023). Analysis of UK median urinary iodine concentrations (UIC) in the *NDNS* showed that all age or sex groups except for women aged 16–49 years met the WHO criteria for adequate iodine status defined as median UIC between 100 and 199 μg/L, and fewer than 20% of the population were below 50 μg/L (OHID 2025). For women of childbearing age, median UIC was 98 μg/L, and 21% were below 50 μg/L and would fail to meet the criteria in pregnancy (median UIC levels between 150 and 249 μg/L). The *NDNS* data do not currently include pregnant women and therefore cannot provide information on the risk of deficiency in this vulnerable group, but it is anticipated that they will be included in the next NDNS fieldwork (2024/25 to 2028/29).

#### 2.2.4 Protein in Older Adults

Older adults are a diverse group, with significant variation in health, physical activity levels, and cognitive status, all of which influence their dietary needs. During the roundtable, discussion focussed on the protein requirements of older adults and the potential implications of transitioning to a more sustainable diet for this population group. NDNS data from a sample of the free‐living general population indicate that average protein intakes meet the reference nutrient intake, RNI (0.75 g protein per kg body weight) in all age/sex groups, although protein intakes show a decline in the 75 years and over age group, where 33% have a lower intake than the RNI (SACN 2021). Additionally, it has been suggested that protein requirements for older adults may be higher (1.0–1.2 g/kg bodyweight per day), compared to younger adults (Bauer et al. 2013). Achieving sufficient protein in this age group can be challenging, particularly in frail older adults and/or those living in residential care or nursing homes, as appetite often decreases with age, poor oral health, dysphagia, cognitive decline, and multimorbidities (Westenhoefer 2005; Borkent et al. 2023). However, adequate protein intake, combined with exercise to enhance muscle protein synthesis, is essential to help mitigate the age‐related loss of muscle mass and strength (Bauer et al. 2013). Both protein quantity and quality (encompassing amino acid composition, bioavailability, and digestibility) are important considerations for muscle health in older adults. The leucine content (an essential amino acid) of foods can further enhance the effect of protein in maintaining muscle mass and strength in older adults (Lixandrão et al. 2021). Typically, animal‐sourced proteins have higher leucine content than plant‐sourced proteins (van Vliet et al. 2015; Morgan et al. 2024), have higher digestibility, and are rich sources of some essential micronutrients. The environmental footprint of food production varies depending on the food source and factors related to how and where it is it being produced, but broadly, plant‐based protein sources can be more environmentally sustainable (Lonnie and Johnstone 2020) and may offer some benefits over animal‐derived protein sources (Alessandrini et al. 2021) (e.g., lower in saturated fatty acids, higher in phytochemicals and fibre). Plant‐sourced proteins in the context of maintaining muscle mass and function to mitigate the development of sarcopenia and protein energy malnutrition in older adults has not been fully elucidated (Morgan et al. 2024). In the absence of a full understanding of the impact of completely transitioning away from animal‐sourced proteins in this vulnerable population group, encouraging older individuals to consume a blend of plant and animal proteins was suggested to be a feasible approach to maintain muscle mass whilst also incorporating more plant‐based proteins in the diet. It is also interesting to note that certain plant/fungal proteins are considered relatively high quality (e.g., soy and mycoprotein) (Hertzler et al. 2020; Derbyshire et al. 2023).

## 3 Policy, Agriculture and Industry Perspectives: Actions to Safeguard Nutritional Security

Following discussion of the key life stages and vulnerable groups which could be most impacted by potential nutrient inadequacies in the environmentally sustainable dietary transition, focus shifted to the food supply chain. Roundtable participants considered the impact of climate change on the agriculture sector and food industry and presented key considerations and potential solutions which could facilitate an equitable transition and prevent existing health disparities from being widened.

### 3.1 Agriculture Perspectives

#### 3.1.1 Impact of Climate Change on the UK Agriculture Sector

Any discussion on the transition to sustainable diets must consider the agricultural sector's dual role in both driving and being vulnerable to climate change and extreme weather events. Agriculture contributes 10%–12% of global GHG emissions (overall food systems contribute around 30%, (Crippa et al. 2021) yet is also highly vulnerable to its impacts (Climate ADAPT 2025)). This sector is integral to the food system, which is currently failing to provide equitable access to healthy and safe diets for the world's population. To ensure future food security and sustainability, urgent action is needed not only to mitigate the environmental damage caused across our food system, but also to build resilience against the effects of climate change. This will likely necessitate low‐carbon farming practices and technologies, properly supported by the UK's governments including providing incentives and addressing barriers for farmers and land managers to diversify land use and management.

#### 3.1.2 Changing Weather Patterns

Changing weather patterns and temperatures, alongside other global disruptors including conflict, threaten food production worldwide, contributing to supply chain disruptions and food price inflation. Those in low‐income and developing countries are most affected, but the impact is also recognised in high‐income countries including the UK. Prolonged periods of drought and heatwaves, followed by bouts of heavy rain, are becoming increasingly common and have major consequences for the agriculture sector (Hanlon et al. 2021). Some direct impacts of weather on farming businesses, from a report assessing impacts of a changing climate on farming and food production in Wales, are summarised in Table 2.

**TABLE 2:** Examples of extreme weather impacts on agriculture.

Impact on farming businesses
Feed and fodder shortages from extreme cold weather Restricted growth of crops and grass from extreme drought or cold weather Livestock deaths from temperature extremes Shortages of drinking water for livestock and irrigation water for crops from droughts Damage to agricultural buildings and other infrastructure from storms Delayed or disrupted milk collections from snowfall and flooding

*Source:* Extreme weather and its impact on farming viability in Wales Farmlytics 2024.

The top 10 warmest years on record have all occurred since 2002 and are becoming the norm (Hanlon et al. 2021). Since 2018, the UK has experienced more intense weather events and patterns, which have severely affected crop yields, food production, and business profits, threatening the long‐term viability of the sector (European Environment Agency 2019; Farmlytics 2024; Met Office 2020; Wheeler and Lobley 2021). For example, a WWF Cymru commissioned report highlighted how drought reduced grass growth in 2018 and 2020, leading to a surge in demand for feed. This in turn drove up prices and caused supply shortages (Farmlytics 2024), negatively impacting cattle and sheep farmers in Wales. Sheep farms were further affected by unseasonally cold weather, increasing losses during the spring lambing season; this was exemplified by ewe numbers dropping from almost 5 million in 2017 to 4.3 million in 2020 (Farmlytics 2024).

Roundtable participants from the agriculture sector discussed the effect of changing weather patterns on crop planting practices and yields as a major concern. Data from the Agriculture and Horticulture Development Board (AHDB 2024) suggest a concerning overall downward trend in UK crop production highlighting the vulnerability of the agriculture sector to climate change. Poor weather has been cited as a reason for the declining size of areas planted with vegetables and vegetable yields (Defra 2024b). The unpredictability of weather events on planting, growing, and harvesting conditions, and the impact on crop yields present a challenging business environment and pose an increasing threat to farmers' livelihoods and the security of the food supply. Amidst the declining volume of fresh domestic produce, reducing pre‐farm gate food waste is also an issue. In 2022, approximately £22 million of produce was wasted in fields due to harvest season labour shortages (National Farmers Union 2022). The Seasonal Worker Scheme, a short‐term visa scheme for migrant farm workers, aims to address labour shortages within horticulture arising from varying seasonal demand (Migration Advisory Committee 2024). In 2025, 43 000 seasonal worker visas are expected to be made available for horticulture. It is important to note that workers under these visas are typically employed seasonally for low wages. Ensuring adequate and fair pay for all workers is important to prevent the exploitation of farm labourers.

Roundtable participants also discussed growing government recognition of the challenges faced by the sector and support through the implementation of policies. The sustainable farming incentive (SFI) was implemented in 2022 (Defra 2020) in an effort to increase farm resilience to climate change. This initiative funded farmers to take environmental land management actions to support more sustainable food production while protecting and enhancing nature; although, unfortunately, it was recently announced that as of March 2025 no new applications will be considered. There is concern that this move might undermine the progress made in enhancing farm resilience and supporting sustainable practices.

#### 3.1.3 UK Food Self‐Sufficiency

The UK food system is highly complex (UKHSA 2023), involving long international supply chains and multiple stages including import and export, industrial processing, storage and transport, retail, food preparation (in households or institutions), consumption and waste management. As an illustration of complexity, processed foods that are produced in one country (with ingredients typically obtained from several countries) may be exported to another country for processing and packaging, and then imported into the UK for consumption. Some foods are also UK‐produced, exported for processing, and later re‐imported. Climate shocks can impact the system at any stage of the supply chain and can be experienced domestically or globally (UKHSA 2023).

To ensure a consistent food supply, the UK relies on a combination of strong domestic production from its agricultural and food manufacturing sectors and a diverse range of international supply sources (Defra 2024a, 2024b, 2024c, 2024d).

The production‐to‐supply ratio, which is a broad measure of national self‐sufficiency, reflects the balance between domestic production and trade. In 2023, the UK's food supply consisted of 62% domestic production for all food and 75% for indigenous foods (those that can be grown in the UK). The remaining 38% of food is sourced through imports (Defra 2024b).

The Eatwell Guide recommends that just over a third of daily food intake should consist of a variety of fruits and vegetables (OHID 2016). The UK is more reliant on imports of fruits and vegetables than for other food categories, producing just 17% and 55% of its supply respectively (Defra 2024b). This is due to factors such as climate, seasonality, and consumer and producer choices. Supply can be affected where imports are from countries vulnerable to climate change and extreme weather, influencing availability and cost, and potentially exacerbating dietary inequalities. For some staple products, such as rice, the UK's climate is unsuitable for cultivation, so it will likely always depend on imports. Yet imports may be from climate‐vulnerable countries that have experienced extreme heat and floods in recent years (Somani 2023). Again, this presents the possibility of future shortages and escalating prices as the frequency and severity of adverse weather events resulting from climate change intensify.

Interestingly, despite the supply chain disruption following Russia's invasion of Ukraine in 2022, which resulted in increased domestic food production costs, food security in the UK is currently described by government as ‘quite stable’. However, increased instability internationally, climate change, and biodiversity loss pose significant longer‐term risks to the security of the UK's food supply (Defra 2024c). With climate and global geopolitical uncertainty, domestic production of affordable food and fresh produce is likely to play a crucial role in the UK's future food security as well as in promoting the health of the population. Disruptions to supply affect food availability, accessibility, cost, and ultimately both nutritional quantity and quality (Fanzo et al. 2021). Such disruptions are likely to disproportionately affect lower‐income groups.

#### 3.1.4 Opportunities for the Farming Sector: Technological Advances

In the latest (seventh) Climate Change Committee carbon budget (CCC 2025), agriculture is highlighted as the fourth biggest GHG emitting sector in the UK. However, by 2040 agriculture is expected to be the second‐highest emitting sector, due to other sectors such as energy having reduced emissions faster. Agricultural emissions have declined very little in the last decade and will need to drop more steeply. Substantial reductions in agricultural emissions can stem from adopting low‐carbon farming practices and technologies, including livestock management measures. Emerging agri‐food research and advancing agricultural technologies may offer opportunities to improve UK food security and meet these challenges, for example through reducing pesticide reliance, enhancing disease and climate resilience and improving the nutrient quality of crops. Following a change in the law, gene‐edited^2^ food can now be developed commercially in England (Genetic Technology (Precision Breeding) Act 2023). Precision breeding technology offers an advantage in the development of resilient crops that will be needed because of climate change. Advances in gene editing also offer promising opportunities for developing bioavailable nutrient‐rich seed variants to counter nutritional deficiencies. As an example, iron deficiency is the most common nutritional deficiency worldwide (Freeland et al. 2024). Cereals are an important dietary source of non‐haem iron but contain absorption inhibitors such as phytic acid that limit its bioavailability. Wheat grains have successfully been gene edited to reduce phytic acid and increase iron bioavailability (Ibrahim et al. 2022). If genetically enhanced seeds like these are integrated into the food chain on a large scale, there is significant opportunity to increase the content and bioavailability of key nutrients available to the population. However, roundtable participants highlighted the need for certain conditions to accelerate the development and acceptance of gene‐edited crops, including market demand, substantial investments from government and industry, and support from non‐governmental organisations.

There are clear challenges for the agricultural industry in transitioning to net zero. Currently, the UK is falling short of achieving the 2050 net‐zero targets for agriculture emissions. In the 2023 Climate Change Committee progress report, attention was drawn to insufficient government focus on promoting sustainable dietary change, inadequate progress in land‐use change targets, gaps in agri‐environment policies and an overreliance on voluntary measures (Climate Change Committee 2023). To be successful, there is a need for more robust, long‐term and mandatory policies that give producers the security and confidence to obtain sufficient investment to produce more nutritious, sustainable and affordable food in the UK while creating jobs and delivering for nature, energy, security and climate‐friendly farming. Any future sustainable farming initiatives need to be multi‐faceted and designed to protect farmers' livelihoods, ensure supply of sufficient affordable nutritious food and minimise environmental damage from agricultural practices. The convergence of technological advancements and government policies aimed at improving sustainable farm management presents opportunities to pave the way to a more sustainable UK agriculture sector in future.

### 3.2 Food Industry Perspectives

As well as the agricultural sector, the food industry is critical to the provision of an equitable, healthier and more environmentally sustainable diet, yet in the current food environment it is failing to do so. The food environment, the combination of the physical, economic, political and sociocultural context in which we buy and consume food (Imperial 2024), fundamentally influences individual dietary choices and impacts on our nutrient intake (Cameron et al. 2016; Atanasova et al. 2022). In turn, the food environment, and what is produced and consumed within it, is shaped by the food industry. Manufacturers, retailers, the out‐of‐home (OOH) sector and delivery apps shape purchasing through mechanisms such as pricing, portion sizing, quality, availability and accessibility of food products, as well as placement and promotion strategies.

Food businesses therefore have an opportunity, and arguably a moral imperative, to improve the food environment for everyone in society to make positive dietary and environmental contributions in the transition to Net Zero. Currently, however, the food environment does not make healthy or environmentally sustainable choices the easiest option for consumers. For example, advertising spending favours foods high in saturated fat, sugars and/or salt, confectionery, snacks and soft drinks over healthier options, such as fruit and vegetables (Goudie 2023).

In the UK, over 95% of the grocery market share is held by the top 10 supermarkets, within which the food sold is predominantly produced by five main manufacturers (UKHSA 2023; Kantar 2024). Households spend just under 80% of their home food budget in supermarkets. However, the OOH sector is an increasingly significant aspect of our interaction with the food environment, with around 20%–25% of calories consumed outside the home (DHSC 2020). Evidence suggests that the food available in the OOH sector is higher in energy, saturated fats, sugar, and salt, and is sold in larger portion sizes compared to the retail equivalents (WHO European Region 2022). Despite the growth of plant‐based options as a whole in the OOH sector, animal‐based food offerings continue to dominate sandwich selections, restaurant menus, and takeaway offerings, often presenting better value (Warner 2024) and making the more environmentally sustainable option less attainable and accessible, especially during the ongoing cost‐of‐living crisis.

#### 3.2.1 The Place of Novel Plant‐Based Alternatives

Sustainable dietary guidelines do not necessitate eliminating meat or other animal‐derived foods entirely but advocate for increasing intake of nutrient‐rich plant foods, including whole grains, fruits, and vegetables, and plant‐based sources of protein such as legumes/pulses, nuts, and seeds. However, the pathway to achieving these dietary changes varies geographically and culturally (Davies et al. 2023), and for some, this diet may include processed plant‐based alternatives (PBAs). These more novel plant‐based food products are designed to mimic and replace animal‐based foods, facilitating their incorporation into habitual diets (Nájera Espinosa et al. 2025; Alae‐Carew et al. 2022). The market for PBAs has grown rapidly over the last decade and may continue as more cost‐effective innovations in alternative proteins are realised. Additionally, innovation in alternatives such as precision fermentation, cultivated/lab meat, and bioprocessing is expected to shape the future of the global food market through the development and commercialisation of new products.

Many people may lack the time or skill to cook from scratch (Lavelle et al. 2016), and processed foods are commonplace within people's diets in the UK (BDA 2021). Thus, PBAs can leverage the familiarity and structure of current meal patterns and convenience and have been recognised as playing a potentially important role in encouraging a shift towards a more plant‐rich diet. Furthermore, PBAs may serve as a stepping‐stone towards diets higher in minimally or unprocessed plant‐based foods. Surveys suggest that PBAs are being used as part of flexitarian diets for those seeking to reduce animal‐based foods, rather than being solely included in vegan diets.

Comparisons between the nutritional quality of PBAs and animal‐based products are complex, as they typically contain or lack different nutrients and therefore may have different impacts on nutritional intakes and status and, potentially, on health outcomes. Reviews of the nutritional composition of PBAs suggest that, on average, they have lower levels of energy and saturated fat, and higher amounts of fibre (El Sadig and Wu 2024). This is of particular note as dietary recommendations for saturated fat and fibre are not being met in all age groups. However, poorly formulated PBAs can contain high levels of salt and have less and poorer quality protein, iron, and vitamin B12 compared to animal products (Bryant 2022; Nájera Espinosa et al. 2025). Not all PBAs should be considered healthier or more sustainable solely because they are ‘plant‐based’; their overall nutritional composition and sourcing must also be considered. It is therefore important to help consumers differentiate within the wide range of plant‐based ‘UPF’ products, to select those with a better nutritional profile (considering saturated fat, salt, fibre, protein, and micronutrient composition). From a nutrition perspective, the shift towards plant‐based substitutes may not pose significant concerns for individuals with balanced, varied diets. However, it could be challenging for vulnerable groups within the population, especially young children and those on lower incomes and/or poor dietary diversity. Hence there is scope for reformulation, for example, to reduce salt content and to improve fortification (Food Foundation 2024).

The group recognised that to encourage broad adoption of a plant‐rich diet, a variety of substitution strategies will likely be required, taking into account key factors such as nutrient profile and bioavailability of the nutrients, cost, accessibility, and convenience. These should include opportunities to champion and more effectively promote plant foods widely recommended in food‐based dietary guidelines such as fruits and vegetables, wholegrains, pulses, and nuts. Consumers may also need support to make healthier choices within the more novel or highly processed plant‐ and fungal‐based food category.

#### 3.2.2 Plant‐Based Alternatives and the Concept of Ultra‐Processed Foods

The role of processed plant‐based alternatives, PBAs in healthier and more environmentally sustainable diets presents an interesting paradox. On one hand, there is a clear need to shift dietary demand towards more plant‐based eating, and, despite their processing, PBAs are generally reported as lower‐carbon alternatives to animal‐based products (Bryant 2022; Coffey et al. 2023; Ritchie 2022; Swanson et al. 2023). On the other hand, these foods, like their meat‐based counterparts (such as sausages, burgers, hot dogs, and other reconstituted meat products), are classified as ‘ultra‐processed’, and health concerns about ultra‐processed foods (UPFs) have never been more prominent (Lane et al. 2024). This topic stimulated much discussion among the roundtable participants, with some holding contrasting views.

There is consistent evidence, largely from observational studies, linking high intakes of UPF (defined by NOVA) with poor health outcomes including heart disease, type 2 diabetes, obesity and cancer, and this is concerning (BNF 2024; SACN 2023b, 2025a, 2025b). However, the SACN statement and rapid review on processed foods and health (SACN 2023b, 2025a) highlights that there continue to be significant limitations in the evidence base, which is almost exclusively observational. It remains unclear to what extent observed associations between ultra‐processed foods and adverse health outcomes are explained by established relationships between nutritional factors and health outcomes. High UPF consumption may be an indicator of unhealthy dietary patterns, characterised by diets that are energy dense, high in saturated fat, salt and/or free sugars, and/or low in fruit, vegetables and fibre. Indeed, many foods and drinks classified as UPF have a poor nutritional profile. In addition, there is some evidence suggesting that certain types of foods classified as UPFs (such as wholegrain low‐sugar cereals, wholemeal breads and some PBAs) may contribute differently to adverse health outcomes, compared to UPFs such as cakes, biscuits and sugar‐sweetened beverages (Cordova et al. 2023). Interestingly, SACN concluded from limited subgroup analyses that the UPF subcategory ‘vegetarian ‐alternative’ products was found to have no association with adverse health outcomes (SACN 2025a). Mechanisms suggested to explain differential effects include energy density, nutrient profile and intake, but good‐quality randomised controlled trials that may help to identify potential mechanisms are needed. Further consideration of subgroups within NOVA groups, particularly the UPF category, and health outcomes may help delineate principal mechanisms.

The UK already has an established nutrient profiling model, HFSS (High in Fat, Sugar, or Salt), to classify the healthiness of foods, which is used in UK regulation (DHSC 2011). Whilst ongoing research is crucial to understand the mechanisms linking processing and health outcomes, the focus from a food industry perspective may be more usefully concentrated on reducing the proportion of energy‐dense, nutrient‐poor HFSS foods (many of which are also UPF) readily available in today's food environment. This can be achieved through reformulation and innovation to create convenient, affordable, desirable products with higher nutritional quality, targeting established nutritional drivers of ill health and reducing economic barriers to healthy food (TLG Hepatology 2025). One important aspect in achieving this is to ensure that reformulation happens with both nutrition and environmental sustainability in focus, which would require a data‐driven strategic approach to navigate the potential ‘trade‐offs’ involved.

#### 3.2.3 Micronutrient Fortification and Plant‐Based Dairy Alternatives

At the roundtable, specific concern was raised around the nutritional quality of some commercially available plant‐based dairy alternatives (PBDAs), particularly for vulnerable groups such as younger children. Whilst plant‐based, calcium‐fortified, unsweetened dairy alternatives are included in UK healthy eating guidelines, there are notable nutrient differences in commercially available PBDAs such as plant‐based alternatives to dairy milk and yogurts. Some non‐organic plant‐based drinks in the UK are fortified with vitamin D, B2 (riboflavin), B12, and calcium (Medici et al. 2023), and some brands/retailer own brands are starting to fortify their drinks with iodine, although this is far from ubiquitous (see earlier nutrients section). Fortification remains variable in the type and quantities of fortificants used across brands, with practices rapidly changing and varying globally, making it difficult to establish the accurate nutrient contribution from these products.

Organic plant‐based drinks are not permitted to be fortified with micronutrients, and yet these may be perceived as superior by consumers with concerns around UPF and a preference for ‘clean label’ products. Plant‐based cheese alternatives are less likely to be fortified than drinks and have varying nutritional profiles depending on whether they are made from nuts and seeds or the cheaper and more widely available varieties based on coconut oil and starch (Craig et al. 2022). Developing a set of nutrient standards to apply to existing and new PBAs has been suggested to inform and harmonise best manufacturing practices (Drewnowski et al. 2021). Currently, the national composition database in the UK (PHE 2021) holds limited data on the range of plant‐based alternatives available on the market. For example, the only plant‐based dairy alternative represented is soy drink. Establishing accurate, up‐to‐date nutritional composition databases is crucial, both to support informed consumer choices and to guide and support the manufacturing of healthier, more sustainable products.

A recent joint report (SACN 2025b) on the benefit and risk assessment of plant‐based drinks (soya, almond and oat), including the consumption of plant‐based drinks by children under 5 years, was produced by the Scientific Advisory Committee on Nutrition and the Committee on Toxicity of Chemicals in Food, Consumer Products and the Environment (SACN/COT). The report concluded that replacing cows' milk with soya, almond or oat drinks could result in potential benefits and potential nutritional risks. For example, such substitutions could lead to lower intakes of saturated fat and higher intakes of dietary fibre and vitamin D (through fortification). However, they may also result in higher intakes of free sugars and insufficient intakes of key micronutrients such as vitamin A and iodine, which is particularly concerning for young children for whom the relative contribution of cows' milk to nutrient intakes is largest (SACN 2025a). Overall, the report found that plant‐based drinks (commonly available in January 2022) were not nutritionally equivalent to cows' milk in relation to both macro‐ and micronutrients. The report noted that an enhanced proposed fortification profile of unsweetened (without free sugars) soya, almond and oat drinks with vitamin A, riboflavin, vitamin B12, calcium and iodine at similar levels to those in cows' milk, and also with vitamin D, would alleviate most of the nutritional concerns related to plant‐based drinks when consumed in place of cows' milk, especially in young children, while retaining the nutritional benefits. However, to date, such comprehensive fortification is not standard in most plant‐based drinks currently available on the market. Furthermore, evidence on the bioavailability of the micronutrient fortificants commonly added to plant‐based drinks compared with that of micronutrients naturally present in cows' milk is limited and this is a research gap. Given the large contributions that cows' milk makes to the nutrient intakes of young children, the greatest concerns with replacing cows' milk with soya, almond and oat drinks relate to this age group, particularly for those who are following a vegan diet.

### 3.3 Policies to Transform the Food Environment

Policies should aim to transform the current obesogenic environment into one where healthier and sustainable options are promoted, readily accessible, and affordably priced (Tobi et al. 2023). Historically, Government policies related to diet and health in the UK have relied primarily on education or information. However, in isolation, this approach has been shown to be ineffective, particularly in a food environment that encourages the purchasing of energy‐dense, nutrient‐poor foods (Mozaffarian et al. 2018; Theis and White 2021). Discussions at the roundtable highlighted the importance of well‐designed policy and regulatory tools in changing the food environment to improve the nation's diet. However, it is crucial that these are universally adopted, typically through mandatory measures, to ensure a level playing field between companies. Consistency in these approaches is also key, as this can provide clarity and inform long‐term innovation and reformulation strategies for businesses. A piecemeal approach, rather than cohesive, whole‐systems policies, may have contributed to the lack of significant progress in improving the current food system (Tedstone 2023). Delays in implementing regulations on promotions have hindered the cultural shift needed for consumers to make healthier and more sustainable choices. Although some retailers have proactively adopted these changes prior to regulation, this is not yet universal (DHSC 2023).

Dietary policies should be designed to encourage and increase consumption of nutrient‐dense, plant‐rich foods like whole grains, fruits and vegetables, pulses, nuts and seeds, which are strongly supported by current evidence for their benefits to both health and environmental sustainability. However, it is important to acknowledge that diets based primarily on ‘minimally processed foods’ have not been effectively promoted within the current food environment and have yet to achieve mainstream appeal. To support a broader transition towards plant‐based eating, there is a need for affordable, convenient, and pragmatic solutions that align with modern lifestyles. In this context, PBAs may have a role to play as part of a wider toolbox for supporting dietary shifts (Table Debates 2024, https://www.tabledebates.org/building‐blocks/nature‐knows‐best). Concerns over UPF may act as a barrier to this transition. Policies should also encourage and incentivise positive reformulation and innovation to improve the nutritional quality of processed plant‐based alternatives by increasing beneficial nutrients such as fibre and ingredients like pulses and vegetables, while reducing levels of saturated fat, salt and sugar. It is important that consumers are supported and guided to choose nutrient‐dense, non‐HFSS, healthier and affordable versions of some processed foods, which may be facilitated by better, clearer labelling. Further research into the health, behavioural and environmental impacts of dietary shifts from animal‐based to plant‐based alternatives could provide valuable insights to support the broader adoption of sustainable diets. It is essential, however, to consider PBAs not in isolation but as integral components of the whole food system.

#### 3.3.1 Voluntary Versus Mandatory Approaches

Recent government policies for the food industry have largely been voluntary, with a primary focus on reducing childhood obesity—an important target, but one not fully integrated with sustainability or dietary inequality. Roundtable participants from the food industry noted that voluntary measures, such as reformulation targets to reduce salt, sugar, and calorie consumption, have received inconsistent support across businesses. Dependent on food category/nutrient of concern, these have been broadly ineffective in leading to any recent significant changes in the nutritional quality of foods. For example, the voluntary sugar reduction programme was launched with an ambition to reduce sugar by 20% by 2020 in the food categories that contribute most to the intakes of children. Only a 3.5% reduction in the sales weighted average total sugar per 100 g in products sold between baseline (2015) and year 4 (2020) was reported, although there were larger reductions for specific food categories, such as 14.9% for breakfast cereals (OHID 2022).

In contrast, roundtable participants perceived the mandatory Soft Drinks Industry Levy (SDIL) as a more successful strategy to reduce sugar in UK diets, suggesting legislative controls and fiscal interventions may be stronger levers to encourage reformulation and healthier portfolios. Sugar‐sweetened beverages have been the primary focus of such taxes globally (WHO 2023a, 2023b), yet the potential impact of taxation of high‐fat, salt or sugar (HFSS) foods to encourage reformulation, balance the price of healthier versus less healthy food items, and constrict sales of unhealthy food across all sectors of the food industry needs more research.

Cost is often cited as a key barrier to healthy eating; thus, using pricing strategies is of particular interest (Corfe 2018; Puddephatt et al. 2020). If unhealthy foods become more expensive via taxes or healthier foods become cheaper through subsidies, the assumption would be that consumers would adjust their purchasing and consumption habits accordingly (Cornelsen et al. 2019). The reality, however, is more complex, as there are difficulties in measuring the impacts from individual food price changes on total diet, as consumers may change their demand for other foods in response (Cornelsen et al. 2019). Nonetheless, understanding price elasticities is essential for assessing how changes in food prices affect consumption. Interestingly, demand for food appears to be more responsive to price increases than to price decreases. Such changes may improve the dietary quality of low‐SES households the most, with the greatest impact seen when the prices of sweet snacks, desserts, and fats/oils increase. Further research is needed to evaluate the comparative effectiveness of price increases versus decreases in improving overall dietary quality at a population level (Cornelsen et al. 2019). There could be a role for fiscal interventions on price, rethinking VAT and subsidies on foods available in both retail and out‐of‐home sectors to facilitate people to eat more sustainably.

#### 3.3.2 Policies for Vulnerable Groups

There is a strong relationship between deprivation and childhood and adult obesity. For vulnerable groups in particular, the UK food environment is not conducive to consistent access to affordable, environmentally sustainable, and healthy food. Evidence suggests that there are more OOH food outlets in deprived areas, and these tend to offer less healthy options (Keeble et al. 2021; Huang et al. 2024). There is also some evidence that exposure to advertisements for HFSS foods and sugar‐sweetened drinks may be higher among children and lower socio‐economic groups (UK Parliament Postnote 2022).

Interventions addressing specific vulnerable groups may be more effective in mitigating dietary and health inequalities. The UK government's Healthy Start Scheme in England, Wales, and Northern Ireland provides valuable payments for low‐income pregnant women and families with children under 4 years to support basic healthy purchases including fruit, vegetables, milk, and pulses (DHSC 2025). In Scotland, Best Start Foods provides payment to pregnant women and families with children under the age of three, who receive certain benefits that can help towards healthy foods like milk, fruit and vegetables, and pulses (Social Security Scotland 2024). Some reports indicate that fruit and vegetable purchases can be increased through voucher support for low‐income families, such as those provided by the Alexandra Rose Charity (https://www.alexandrarose.org.uk/). Similarly, the number of fruit and vegetable portions purchased per customer has significantly increased in stores participating in a retailer top‐up initiative for Healthy Start Vouchers on fresh, canned, or frozen fruit and vegetables. However, it should be recognised that the UK government's Healthy Start Scheme has lower than expected take‐up rates; addressing key issues such as offering support during the (sometimes lengthy) application process would improve take‐up and help those who need it (Sustain 2023; Barrett et al. 2024).

When discussing consumer behaviour change, there are often underlying assumptions that people have the power (resources and environment) to choose to change their behaviour. This may not be true for lower‐income groups as cost, accessibility, and lower personal agency may be barriers to these groups adopting healthier or more sustainable food patterns (Briazu et al. 2024). Further price interventions similar to the Healthy Start Scheme could play a valuable role in overcoming some of these barriers and supporting lower‐income groups to eat more sustainably. Understanding the interactions between foods, dietary behaviours, and specific needs with seldom‐heard communities will be crucial in addressing the unique challenges faced by different populations(Hanson et al. 2024). The *FIO/DIO Food* multi‐collaborative project (https://www.abdn.ac.uk/rowett/research/fio‐food/index.php) aims to address dietary inequalities among individuals living with obesity and food insecurity by improving access to environmentally sustainable and healthier food choices in the retail environment.

#### 3.3.3 Evidence for Effective Interventions

A recent systematic review (Wadi et al. 2024) investigating interventions that promote environmentally sustainable diets in high‐income countries through behaviour change models concluded that evidence supports the use of multicomponent interventions through education, persuasion, and environmental restructuring to provide opportunity for change. However, little high‐quality research was available, and more robustly designed intervention studies are needed to inform future guidelines and policies, and a better understanding of effective strategies to increase sales of healthier and more sustainable foods. Existing trials have suggested some approaches with varying degrees of success (see Table 3), including experimenting with pricing, promotions, and positioning to influence purchasing behaviours. As an example, a 4‐week retailer price promotion on fruit and vegetables showed a 78% uplift in January 2020 and a 56% uplift in January 2021, although this was not consistent across all products; sales of lower‐value or less familiar items, such as swede, did not change. A review to evaluate interventions within major UK grocery stores found that some interventions, including increased availability and promotions, were associated with short‐term changes in food purchasing behaviours in the desired direction, but strategies that solely aim to inform customers about healthier options are unlikely to work in isolation. The impact of promotions on consumer behaviour may diminish over time and may not be sustainable for retailers in the long term (Piernas et al. 2022). In the Scottish OOH sector, Public Health Scotland, in partnership with Food Standards Scotland, developed a voluntary Eating Out, Eating Well Framework (EOEW) and a children's menu code of practice (COP) to support businesses to provide and promote healthier options. The principles for menu improvement include increasing fruit, vegetables, fibre, and oil‐rich fish, reducing fat, salt, and sugar, and taking actions on promotions, availability, portion size, calories, and food produced in a sustainable manner. The EOEW and COP are being piloted by a range of food outlets throughout 2024, which will generate insights on their impact, informing the next steps.

**TABLE 3:** Summary of trials carried out in the UK to improve the food environment.

Mechanism	Trial	Sector	Impact	Conclusion
Pricing and promotion	Promoting 3 different packs of fresh fruit and vegetables (May–November 2019)^1^	Retailer	13% net increase in sales	Price promotions that make healthier food more affordable can increase sales of these products.
	Price and child‐focused character promotions on healthier products (August–October 2019)^1^	Retailer	72% rise in sales of lower sugar baked beans; 387% rise in sales of fruit	The use of family‐friendly characters to incentivise and reward the purchase of healthier products, in combination with price promotions, can have significant impact on sales, but it is unclear of the specific impact of the children's character promotion.
	Personalisation, gamification and rewards on vegetables through loyalty app (4 weeks, 2021)^2^	Retailer	Fruit and veg increased by 3.6 portions per shopper per week	Incentivisation and reward tactics can drive meaningful changes.
	Healthy Start vouchers were supplemented by retailer by £2 (26 weeks, 2021)^3^	Retailer	In subset analysis of families living in Yorkshire and the Humber, FV portions purchased per household increased by an average of 0.9 portions per day (Thomas et al. 2023)	Promotions targeting low‐income families can help increase fruit and veg intake.
	Reducing the price of different fruit and veg to 60p, placed prominently alongside an advertising campaign (4‐week periods in January 2020 and January 2021)^4^	Retailer	Promoted fruit and veg increased by average of 78% (2020) and 56% (January 2021). This increase occurred over the first 3 weeks. Sales declined in the 4th week.	Price is a major barrier to the purchasing of healthy food. Removing this barrier to make fruit and veg more affordable may make fruit and veg more accessible and increase sales in the short term.
Availability	Lower fat (< 4%) frozen chip alternative introduced with 53% fat and 13% calorie reduction (January–September 2019)^1^	Manufacturer	19% net decrease of sales of less healthy chips	Food companies can shape demand, introducing healthier alternatives in‐store could shift purchasing towards healthier options and decrease the sales of less healthy alternatives.
	Introduction of healthier, in place of less healthy, biscuit options (May–August 2019)^1^	Retailer	No significant impact	Switching less healthy biscuits for healthier ones could have a positive impact but retailer data found consumers switched to different categories—larger scale trials are needed.
Availability	Increase availability of healthier versions of popular foods (e.g., low and no calorie soft drinks, wholemeal bread, fruit and healthier confectionery) (2020–2022)^5^	35 independent convenience stores (Southwark Pilot)	Increased availability and sales of healthier products	Convenience store owners were open to change and, with support from experts, increasing availability of healthier options could drive sales and profits.
Positioning	Less healthy breakfast cereals moved out of eye‐line, high‐fibre, low sugar cereals moved to eye‐level (May–August 2019)^1,2^	Retailer	No significant impact	Positioning changes within aisles may need to be bolder and combined with pricing and promotional strategies to increase prominence and impact.
	Positioning plant‐based meat alternatives in a separate meat‐free bay with promotional messaging within the meat aisle (2021)^6^	Retailer	Sales of equivalent meat‐free products increased by approximately +31% (compared to 6% in the control stores)	Prominent positioning of meat‐free products into the meat aisle in a supermarket successfully increased sales of meat‐free alternatives in the longer term but was not effective in reducing sales of meat products.
	Placing plant‐based alternatives (e.g., sausages) immediately beside meat equivalent in the meat aisle (2021)^7^	Retailer	Reduced sales of plant‐based products by 30%	Placing plant‐based meat alternatives beside their meat equivalents, rather than in a separate bay within the meat aisle, can highlight higher price difference of plant‐based alternatives.
	Seasonal chocolate confectionery moved out of prominent displays (March–April 2019)^1^	Retailer	22% net reduction in purchases	Removing less healthy products from prominent displays can have a positive impact. Greater regulation to ensure parity across businesses could mitigate commercial risks of such interventions.
	Shelf labels highlighting healthier soft drinks (May–August 2019)^1^	Retailer	No significant impact	Shelf labels in isolation did not appear to be enough to shift purchasing behaviour. The message on the labels may also determine impact.
Social feedback	Shelf labels using social feedback to nudge healthier options (3‐week trial)^1^	Retailer	No significant impact	Providing social feedback alone may not be enough to shift purchasing habits. Other methods of providing this feedback with more obvious positioning may be more effective.

*Source:* ^1^Consumer Goods Forum. 2020. ^2^BNF 2023. ^3^Sainsbury's. 2022. ^4^IGD 2021. ^5^IoUH 2020. ^6^Piernas et al. 2022; ^7^IGD 2022.

## 4 Collaborations and Solutions

Multi‐disciplinary collaboration and well‐funded research partnerships between the food industry, the scientific community (with a focus on behavioural, environmental, nutrition and food sciences), policymakers and NGOs are essential for developing innovative, real‐life solutions to major dietary, health and sustainability challenges. Such collaboration will also help bridge the gap between research and practical implementation. Data sharing, collaboration and long‐term strategies are crucial for integrating sustainability and health across food systems. Retailers and manufacturers would benefit from a well‐recognised, measurable definition of what constitutes ‘sustainable’ food products. Developing stronger, long‐term relationships between retailers/manufacturers and producers could allow supplier standards for nutritional and environmental criteria to be established, including enhancing biodiversity. Additionally, both regulation and incentivisation are critical to businesses reshaping their total portfolios, rather than simply introducing a limited number of healthy options. Roundtable participants considered that offering premium payments to producers who meet these standards could encourage them to adopt better practices. Such long‐term partnerships can provide the assurance and support needed for producers to make, and invest in, the necessary changes to progress towards more sustainable and healthier portfolios.

Roundtable participants discussed the role of external stakeholders and investors in encouraging the food industry to develop healthier product portfolios and urging the government to implement progressive policies. This includes the important influence that NGOs and investors can have in holding companies accountable and driving meaningful change.

The importance of data was also highlighted as essential for monitoring and incentivising progress. The UK Government's voluntary Food Data Transparency Partnership (FDTP) (https://www.gov.uk/government/groups/food‐data‐transparency‐partnership), which aims to improve the availability, quality, and comparability of data across the food supply chain and drive positive change towards more environmentally sustainable and healthier food and drink production and sales, was mentioned as an example of data strategies. Roundtable participants expressed disappointment over the voluntary nature of this data provision, as the original plan proposed a mandatory approach requiring large businesses to report publicly against a consistent set of health and sustainability metrics. There have also been calls for corporate protein disclosure, reporting on progress in protein diversification, with a focus on rebalancing animal and plant protein sales (WWF 2022).

The roundtable participants acknowledged the complexity of transforming the food system, shifting eating behaviours and consumption, production and retail structures, whilst considering the interplay between health, environment and socioeconomic factors. Despite business commitments to improving health, nutrition and the environment, there is a need for more substantial changes in the food industry. Sustainable and healthier dietary shifts often compete with business profitability (White et al. 2020), and whilst commercial challenges must be recognised and addressed, meaningful progress requires the industry to undertake multiple actions simultaneously.

## 5 Key Points From the Chair

In summing up, the Chair reflected on a number of key points, including concerns about whether the transition to more environmentally sustainable diets could negatively impact or exacerbate existing inequalities. Whilst acknowledging the urgent need for climate mitigation in food systems, there is concern about potential unintended social, environmental, economic and health consequences, which could disproportionately affect the most vulnerable populations, both nationally and globally. It is crucial that this transition does not exacerbate existing dietary inequalities, particularly among nutritionally vulnerable groups, such as those living with food insecurity, young children and older adults. Achieving this will require a collaborative approach involving public, private and civil society organisations across the food system.

UK dietary survey data provides evidence of poor dietary patterns and risk of inadequate intake of some micronutrients, including calcium, iodine, iron, and zinc in some population groups. Meat and dairy products are typically important sources of these nutrients in the current diet. As the shift to more sustainable diets involves reducing animal‐based foods, it is crucial to ensure that appropriate nutrient‐dense alternatives are available and accessible, particularly for nutritionally vulnerable groups. Other important nutrition considerations discussed included protein quality, focusing on amino acid profiles and digestibility. While protein intake is generally sufficient for most of the UK population, in older people, whose needs may be higher yet intake often lower, both protein quantity and quality become more important.

Protein was also considered in the context of the novel alternative protein sector where innovation is aiming to find environmentally sustainable solutions through algae, fungi, bacteria, aquatic plants, precision and biomass fermentation, cultivated meat and novel aquaculture. Advances in science and technology hold substantial potential to enhance the sustainability, health, equity and resilience of global food systems. Yet these innovations may also present significant trade‐offs, along with regulatory and ethical concerns. As the sector grows and technologies are increasingly translated into new products and processes with diverse sources, it is essential to remain mindful of potential health risks, such as micronutrient inadequacies related to concentration present and/or poorer bioavailability, as well as ensure consumer acceptance. Moreover, policy initiatives should focus on the whole diet, considering broader socioeconomic factors, rather than adopting a reductionist approach centred on individual food groups, products or nutrients. A holistic approach to improving diet quality and shifting towards healthier, more sustainable dietary patterns should be central to these efforts.

The Roundtable also explored the impact of the concept of UPFs on the discussion of healthier and more sustainable diets. This discussion was in part because the transition to more plant‐rich diets is likely to be facilitated by plant‐based alternatives, many of which fall under the NOVA Group 4 classification (Monteiro et al. 2019). Although there is consensus from epidemiological studies that high intake of UPFs correlates with poorer health, the challenge lies in understanding the underlying reasons for this association and whether the association is causative. Strong evidence links energy‐dense diets, dominated by foods categorised as HFSS and low in fruits and vegetables and fibre, to an increased risk of obesity, chronic diseases such as heart disease and type 2 diabetes, and certain types of cancer. However, it is important that the debate surrounding UPFs should not detract from efforts to reduce consumption of HFSS foods. Instead, this discussion should be used to refocus attention on transforming the food system and driving a shift towards dietary patterns that are both sustainable and support health.

Public procurement may play a crucial role in this shift and serve as a powerful lever for transforming the food system. Public institutions (such as schools, hospitals and care homes, prisons and government offices) which provide meals to diverse communities, can influence the food supply chain through sustainable food procurement strategies. This approach shifts the focus away from relying solely on individual consumer choices and to a more provision‐centred model that influences consumption patterns, without placing the all the onus on individuals to change their behaviour.

Indeed, consumer behaviour in the context of sustainable diets is complex and poorly understood, with sociodemographic variables often influencing individuals' agency to change. A greater understanding of behaviour may be facilitated by utilising consumer data to explore how the food environment impacts on behaviours and affects health and wellbeing. This insight could include utilising retail data from shopping transactions and loyalty cards to analyse the interplay between health, sustainability, and cost, as well as testing interventions aimed at promoting healthy and more sustainable diets. Real‐life consumer behaviour research could also guide more effective communication strategies to help shift behaviours in support of healthier and more sustainable choices.

The roundtable participants acknowledged the significant role the private sector (manufacturers, retailers and out‐of‐home providers) plays in shaping the food environment and emphasised the need for accountability. Whilst there is recognition of some genuine desire for change in the food industry, concerns were raised that voluntary actions alone will not be sufficient to transform the food system. Well‐designed regulatory policies and mandatory reporting could create a level playing field for the industry, enabling it to influence consumer choices and behaviours more effectively.

On the supply side, agriculture plays a crucial role in achieving improved environmental outcomes, net zero emissions and global food security. Agriculture has a significant impact on climate change, while also being highly vulnerable to it. The sector is experiencing, and will continue to experience, significant challenges as climate change effects intensify. There are no simple solutions, and improving resilience will require a range of measures and subsidised policy initiatives to enhance productivity, efficiency and land management. The key question is how to implement such policies cohesively to ensure they are both climate and nutrition orientated.

In the Chair's concluding remarks, it was emphasised that climate change is an urgent issue that demands large‐scale change. The Chair called for more interdisciplinary and innovative thinking among nutrition and climate change scientists. The challenge presented to the nutrition science community was clear; if there are concerns about how the environmental sustainability and climate mitigation adaptation agendas are advancing, potentially with unintended consequences for nutrition and food security, nutritionists must engage actively in those discussions. They need to sit at the climate table to ensure that nutrition needs are met, particularly for the most vulnerable within populations.

## Author Contributions

A.S., L.B., S.S. and M.H.T. contributed to the conception of the RT and paper, and the analysis and interpretation of scientific literature for the work; A.S., L.B. and Z.H. drafted the work, A.S., S.S., M.H.T. and J.F. revised it critically for important intellectual content; A.S., L.B., S.S., M.T. and J.F. had final approval of the version to be published.

## Conflicts of Interest

Funding to support the British Nutrition Foundation's charitable aims and objectives comes from a range of sources including membership, donations and project grants from food producers and manufacturers, retailers and food service companies; contracts with government departments; conferences, publications and training; overseas projects; funding from grant‐providing bodies, trusts and other charities. Further information about the British Nutrition Foundation's activities, funding and governance can be found at https://www.nutrition.org.uk/aboutbnf/.

## Data Availability

The authors have nothing to report.

## Endnotes

^1^
SACN, Scientific Advisory Committee on Nutrition, **is** the independent advisory body on nutrition and related health matters to UK government.

^2^
Gene editing refers to a set of processes where targeted changes (removal or altering) are made to a plant's DNA. These processes mimic those that naturally occur within traditional breeding.
